# Pinch‐and‐Punch Excision: A Minimally Invasive Technique for Idiopathic Scrotal Calcinosis—A Case Report

**DOI:** 10.1155/criu/6621943

**Published:** 2026-04-28

**Authors:** Krittin Naravejsakul

**Affiliations:** ^1^ Department of Surgery, School of Medicine, University of Phayao, Phayao, Thailand, up.ac.th

**Keywords:** aesthetic urologic surgery, biopsy punch excision, case report, histopathology, idiopathic scrotal calcinosis, minimally invasive surgery, sutureless technique

## Abstract

**Introduction:**

Idiopathic scrotal calcinosis (ISC) is a rare, benign condition characterized by painless, calcified nodules within the scrotal dermis. Although typically asymptomatic, progressive growth and cosmetic concerns often lead patients to seek surgical intervention.

**Case Presentation:**

We report a 35‐year‐old male with multiple asymptomatic scrotal nodules progressively enlarging over 3 years. Physical examination revealed firm, subcutaneous nodules ranging from 2 to 20 mm. Laboratory investigations, including serum calcium, phosphate, and parathyroid hormone, were unremarkable. Histopathological examination confirmed the diagnosis of ISC, revealing basophilic calcified deposits within a fibrous stroma without cystic epithelial lining, consistent with dystrophic calcification.

**Intervention:**

A “pinch‐and‐punch” excision technique was performed under regional anesthesia supplemented by tumescent local infiltration. Individual nodules were elevated by pinching the overlying scrotal skin, followed by targeted removal using 2–4 mm disposable biopsy punches. No sutures were required.

**Outcome:**

The procedure was completed with minimal bleeding and no intraoperative or postoperative complications. Complete epithelialization occurred within 1 week via secondary intention. At 3‐month follow‐up, no recurrence was observed and the patient reported high satisfaction with the aesthetic outcome.

**Conclusion:**

The pinch‐and‐punch excision technique—distinguished by its combination of the tissue‐elevation pinching maneuver, tumescent infiltration for hydrodissection, and sutureless wound management—is a safe and cosmetically favorable approach to ISC, particularly for patients with multiple nodules. Histopathological confirmation remains essential. Further prospective studies with longer follow‐up are warranted.

## 1. Introduction

Idiopathic scrotal calcinosis (ISC) is a rare, benign dermatological condition characterized by multiple, painless, calcified nodules localized within the scrotal skin. The condition was first described by Lewinski [1] and has since been reported sporadically in the medical literature, with approximately 1000 cases documented worldwide.

ISC typically presents in early adulthood and is often asymptomatic. However, some patients experience pruritus, secondary infections, or spontaneous extrusion of calcified material, leading to local irritation and discomfort. Although ISC follows a benign course with no known risk of malignant transformation, the progressive enlargement and increasing number of lesions often prompt affected individuals to seek surgical intervention, primarily for cosmetic or symptomatic relief.

The pathogenesis of ISC remains a subject of debate. The most widely accepted theory proposes that dystrophic calcification occurs secondary to the degeneration or rupture of epidermoid cysts, with histopathological findings revealing dermal nodules containing basophilic calcified deposits surrounded by giant cell granulomas, indicating a foreign body‐type inflammatory response. Alternative theories suggest idiopathic degeneration of the dartos muscle or ectopic calcium deposition, although these mechanisms lack substantial scientific validation. ISC is not associated with systemic metabolic abnormalities; affected individuals consistently demonstrate normal serum calcium, phosphorus, and parathyroid hormone levels.

In Thailand, reported cases of ISC are limited, with only a few case reports available, suggesting that the condition may be underrecognized or underreported in this region. Surgical excision remains the primary treatment modality. Traditional techniques such as elliptical or fusiform excisions are effective but may result in prolonged operative time, extensive scarring, and challenges in maintaining scrotal skin tension. Various minimally invasive approaches have been explored, including punch‐based excision methods for scrotal lesions.

In this case report, we describe a “pinch‐and‐punch” excision technique for ISC that integrates a tissue‐elevation pinching maneuver with tumescent anesthesia and sutureless wound management. We detail histopathological confirmation of diagnosis, operative steps, and three‐month follow‐up outcomes, and discuss how these elements together distinguish our approach from previously described punch excision methods.

## 2. Case Presentation

**Figure 1:**
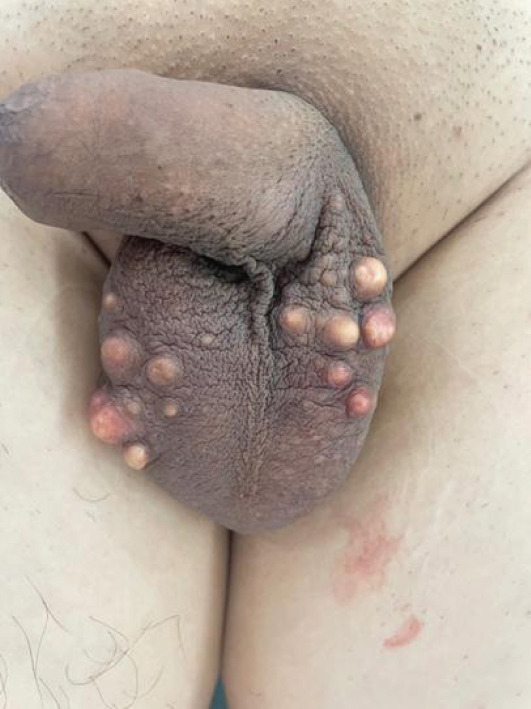
Preoperative image showing multiple firm, subcutaneous nodules within the scrotal skin, ranging from 2 to 20 mm in diameter. The overlying skin is intact with no signs of ulceration or discharge.

A 35‐year‐old male, with no significant past medical or surgical history, presented to the urology outpatient department with progressively enlarging scrotal nodules that had increased in both size and number over the past 3 years. On physical examination, multiple painless, firm, subcutaneous nodules were observed within the scrotal wall, ranging from 2 to 20 mm in diameter. The patient denied any history of pruritus, ulceration, chalky discharge, scrotal inflammation, infection, or trauma. There was no personal or family history of metabolic disorders, and no other cutaneous lesions were identified upon general examination (Figure 1).

Laboratory investigations, including serum calcium, phosphorus, and parathyroid hormone levels, were within normal limits, ruling out systemic metabolic disturbances.

Surgical excision was performed under spinal anesthesia. All excised specimens were sent for histopathological examination. Histopathological analysis revealed multiple dermal nodules composed of basophilic calcified material within an acellular fibrous stroma. No cystic epithelial lining was identified. Scattered chronic inflammatory cells and foreign body‐type giant cells were present in the periphery of the calcified deposits, consistent with a dystrophic calcification pattern. These findings confirmed the diagnosis of ISC.

## 3. Surgical Technique

Following standard antiseptic preparation, local anesthesia was administered using a tumescent solution composed of 1% lidocaine with 1:100,000 adrenaline, infiltrated around each lesion via a 30‐gauge needle. This approach offers several advantages: reduced intraoperative bleeding through vasoconstriction, decreased systemic lidocaine toxicity due to slow absorption, effective perilesional pain control, and a hydrodissection effect that facilitates the separation of calcified nodules from the surrounding connective tissue and superficial dartos fascia. Gentle palpation and rubbing of each nodule following infiltration further loosened the calcified deposits within the subcutaneous plane. A waiting period of approximately 10 min ensured optimal vasoconstriction, providing a near‐bloodless operative field.

The key technical maneuver involved pinching—rather than stretching—the overlying scrotal skin to elevate and stabilize individual nodules prior to punch application. This pinching motion serves to: [2] highlight and localize the subcutaneous nodule beneath the skin surface; [3] limit the effective depth of punch penetration, thereby reducing the risk of deep tissue perforation; and [4] generate pressure that assists in the spontaneous extrusion of the nodule through the punch incision.

**Figure 2:**
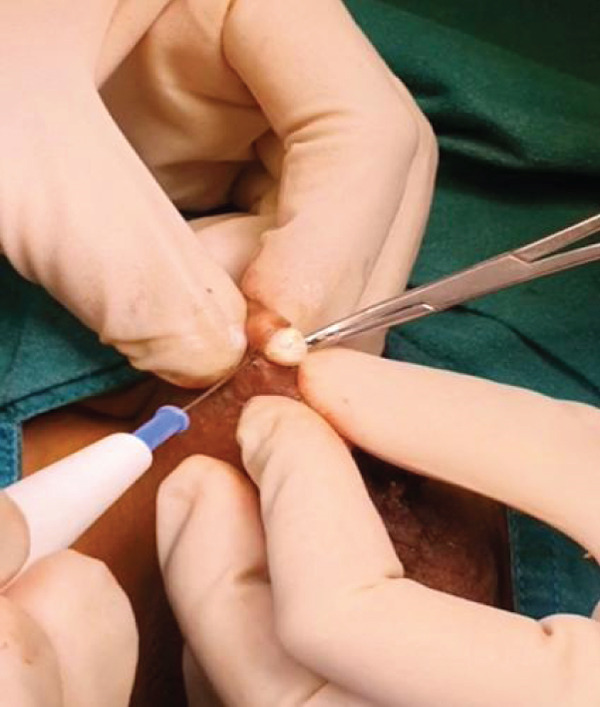
Intraoperative demonstration of the pinch‐and‐punch technique. The scrotal skin is pinched to highlight and stabilize the subcutaneous nodule before applying a 2–4 mm disposable biopsy punch. This maneuver facilitates controlled punch depth and allows spontaneous extrusion of the lesion.

Depending on lesion size, a 2–4 mm disposable biopsy punch was applied over each elevated nodule (Figure 2). Small nodules were retrieved with fine forceps following punch incision. Larger lesions (> 4 mm) that did not spontaneously extrude were first punctured to express their chalky contents, after which any residual calcified material or fibrous wall was grasped with forceps or a mosquito hemostat and removed. Small scissors were used selectively for residual fibrous tissue dissection.

**Figure 3:**
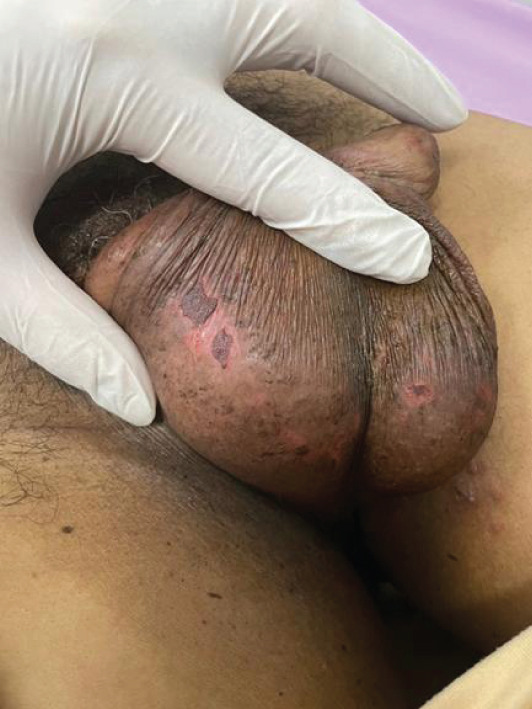
Postoperative image at Day 7, demonstrating complete epithelialization of all excision sites with minimal scarring. Healing was achieved by secondary intention without suture application.

Following excision of all identifiable nodules, wounds were dressed with topical antibiotic ointment. Sutures were not applied, as the combination of tumescent‐induced hemostasis and the contractile nature of the scrotal skin rendered the small punch wounds amenable to healing by secondary intention. Avoidance of sutures also reduced operative time, procedural cost, and the risk of needlestick injury. Complete wound epithelialization was observed within 1 week postoperatively (Figure 3).

## 4. Discussion

ISC is a rare benign condition characterized by multiple asymptomatic calcified nodules localized to the scrotal skin. Its exact pathogenesis remains uncertain, with prevailing theories suggesting dystrophic calcification secondary to the degeneration or rupture of epidermoid cysts, despite the absence of systemic calcium‐phosphorus metabolic abnormalities. Other less substantiated hypotheses include idiopathic degeneration of the dartos muscle or primary ectopic calcium deposition. ISC predominantly affects males in early adulthood, though its true prevalence remains underreported due to its asymptomatic nature and the reluctance of affected individuals to seek medical attention.

Histopathological confirmation is fundamental to establishing the diagnosis of ISC, as the clinical presentation overlaps with epidermoid cysts and other scrotal calcified lesions. In the present case, pathological analysis demonstrated basophilic calcified deposits within a fibrous stroma, with no cystic epithelial lining and surrounding foreign body‐type giant cells—findings consistent with dystrophic calcification and confirming an ISC diagnosis. Authors reporting on ISC should routinely submit all excised specimens for histopathological examination.

The primary indication for intervention is cosmetic concern or the development of symptoms such as pruritus, secondary infection, or extrusion of calcified material. Various surgical techniques have been described for ISC management, ranging from traditional elliptical excision to minimally invasive approaches. Although elliptical excision effectively removes lesions, it is time‐consuming and may result in extensive scarring, prolonged wound healing, and difficulty maintaining optimal skin tension. Punch‐based excision techniques [5, 6]for scrotal cysts and ISC have been previously reported by Al Aradi et al. [7] and Zhou et al. [8].

The technique described in the present report builds upon these prior descriptions and integrates three specific elements that, in combination, differentiate it from previously reported approaches: [2] the *pinching maneuver*—actively elevating and stabilizing the skin fold over the target nodule rather than stretching the scrotal skin flat—which enhances nodule localization and limits punch depth; [3] *tumescent infiltration* providing a hydrodissection plane [9] that loosens calcified deposits from surrounding connective tissue and provides near‐bloodless conditions; and [4] intentional *sutureless wound management* exploiting the contractile properties of scrotal skin to achieve rapid second‐intention healing. We acknowledge that each of these elements has been applied individually in various contexts; however, their systematic combination and the specific pinching technique have not been explicitly described as a unified operative approach for ISC in the available literature.

With respect to follow‐up, the patient was evaluated at 1 week and 3 months postoperatively. At the 3‐month review, all excision sites demonstrated complete healing with minimal residual scarring. No recurrence of calcified nodules was detected on clinical examination. The patient reported high satisfaction with both the functional and cosmetic outcomes. Longer‐term follow‐up data are not yet available; however, we acknowledge that ISC may recur if subclinical lesions are present at the time of initial surgery, and extended surveillance is recommended for all patients.

Limitations of this report include the single‐case design, the absence of standardized patient‐reported outcome measures, and the relatively short follow‐up duration. Further prospective studies with larger sample sizes and long‐term follow‐up are needed to validate the efficacy, recurrence rate, and cosmetic superiority of this approach compared with traditional excision methods.

## 5. Conclusion

ISC is a rare but benign condition presenting with multiple painless scrotal nodules. Histopathological confirmation of diagnosis is essential and should be performed in all cases. Surgical excision remains the treatment of choice for symptomatic or cosmetically concerning cases. The pinch‐and‐punch excision technique—incorporating a tissue‐elevation pinching maneuver, tumescent hydrodissection, and sutureless wound healing—offers a practical, minimally invasive approach with favorable cosmetic outcomes. At 3‐month follow‐up in this single case, no recurrence was observed. Further studies with larger sample sizes and extended follow‐up are warranted to fully evaluate the long‐term efficacy and recurrence profile of this approach.

## Author Contributions

K.N.: conceptualization, surgical intervention, data collection, writing—original draft, writing—review and editing, and final approval.

## Funding

No funding was received for this manuscript.

## Disclosure

All authors have read and approved the final version of the manuscript. Krittin Naravejsakul had full access to all of the data in this study and takes complete responsibility for the integrity of the data and the accuracy of the data analysis.

## Ethics Statement

This case report was reviewed and approved by the Institutional Review Board of the University of Phayao (Approval Number: HREC‐UP‐HSST 1.1/042/68). The study was conducted in accordance with the principles of the Declaration of Helsinki.

## Consent

Written informed consent for publication of this case report, including clinical photographs, was obtained from the patient.

## Conflicts of Interest

The author declares no conflicts of interest.

## Data Availability

The data supporting the findings of this case report are available from the corresponding author upon reasonable request, subject to applicable privacy regulations.
